# Calibrating spatiotemporal models of microbial communities to microscopy data: A review

**DOI:** 10.1371/journal.pcbi.1010533

**Published:** 2022-10-13

**Authors:** Aaron Yip, Julien Smith-Roberge, Sara Haghayegh Khorasani, Marc G. Aucoin, Brian P. Ingalls

**Affiliations:** 1 Department of Chemical Engineering, University of Waterloo, Ontario, Canada; 2 Department of Applied Mathematics, University of Waterloo, Ontario, Canada; Fudan University, CHINA

## Abstract

Spatiotemporal models that account for heterogeneity within microbial communities rely on single-cell data for calibration and validation. Such data, commonly collected via microscopy and flow cytometry, have been made more accessible by recent advances in microfluidics platforms and data processing pipelines. However, validating models against such data poses significant challenges. Validation practices vary widely between modelling studies; systematic and rigorous methods have not been widely adopted. Similar challenges are faced by the (macrobial) ecology community, in which systematic calibration approaches are often employed to improve quantitative predictions from computational models. Here, we review single-cell observation techniques that are being applied to study microbial communities and the calibration strategies that are being employed for accompanying spatiotemporal models. To facilitate future calibration efforts, we have compiled a list of summary statistics relevant for quantifying spatiotemporal patterns in microbial communities. Finally, we highlight some recently developed techniques that hold promise for improved model calibration, including algorithmic guidance of summary statistic selection and machine learning approaches for efficient model simulation.

## 1. Introduction

Microbial communities are ubiquitous [1]. They are responsible for life-sustaining planetary processes [2,3], and they maintain health in almost all metazoans, including humans [4]. Humanity has a long history of harnessing the power of natural microbial communities in, e.g., food fermentation [5], waste water treatment [6], and health [7]. Advances in sequencing and omics technologies have elucidated the roles of individual microbes within their communities, and how they contribute to the overall community function. This, in turn, has opened opportunities for manipulating and designing microbial communities to perform useful tasks across the bioeconomy [8].

Within microbial communities, species interact through, e.g., physical contact, competition for nutrients, metabolite exchange, toxin production, antibiotic inactivation, and quorum sensing. These interactions are shaped by a multitude of factors such as evolution [9] and abiotic features of the environment [10]. The network of interactions determines species abundances in a microbial community, thereby influencing the community’s operation [11–13]. Growth of most communities involves attachment, and so cell–cell interactions influence the community’s spatial arrangement [14]; the spatial structure may in turn influence the evolution of cooperative or competitive interactions [15,16]. To complicate things further, the community composition can also be impacted by the environment’s colonization history [17,18]. The complex dependencies among cellular interactions, spatial dynamics, evolution, and community function make precision manipulation of microbiomes difficult. Mathematical models can be used to address this challenge by untangling the factors governing community behaviour.

**Fig 1:**
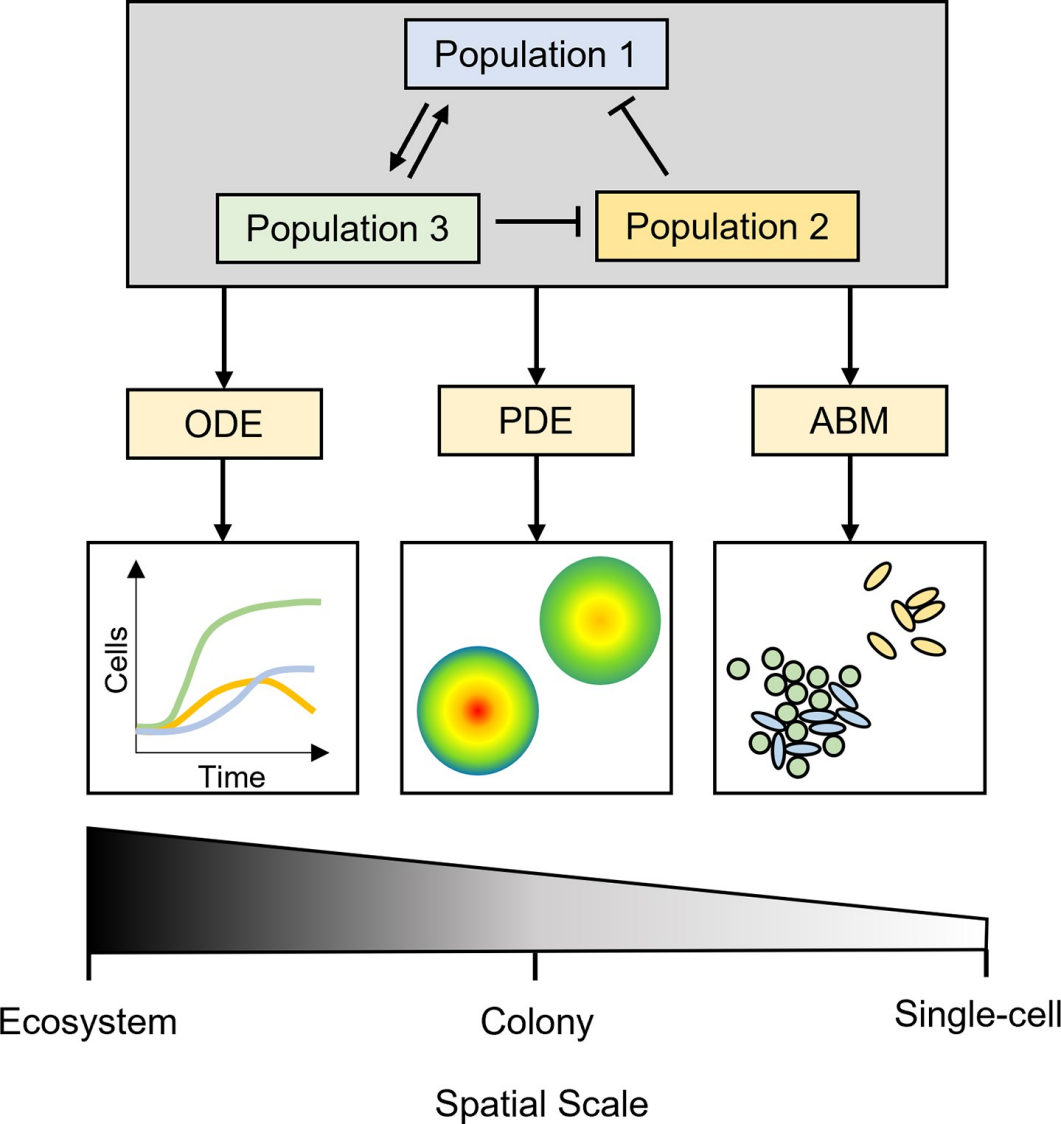
Modelling frameworks commonly used for capturing the behaviour of microbial communities, with associated spatial scales. ABM, agent-based model; ODE, ordinary differential equation; PDE, partial differential equation.

To engineer microbial communities to suit our needs, we must first acquire a thorough understanding of how these communities operate [19]. Mathematical models can be used to guide rational manipulation and design of microbial communities, to predict how communities will behave, and to determine how well they will perform desired functions in, e.g., biotechnology, health and medicine, food and agriculture, and energy production [8]. Fig 1 depicts 3 classes of predictive models commonly used to describe microbial communities: ordinary differential equations (ODEs), partial differential equations (PDEs), and agent/individual-based models (henceforth referred to as ABMs) [20,21]. The primary distinction between these model types is their spatial resolution. ODE models are built on the assumption that the dynamics are not dependent on spatial distribution. Consequently, simulation and analysis of these models incurs a relatively low computational cost. PDE models explicitly account for spatial distribution, but describe local averages, rather than individuals. In contrast, ABMs can capture the spatiotemporal behaviour of individuals within populations, and so can account explicitly for heterogeneity among cells. This high degree of resolution comes at substantial computational cost, which scales (potentially nonlinearly) with the number of individuals and interactions in the population. ABMs are often combined with PDE and ODE submodels to describe phenomena such as intracellular biomolecular network dynamics and extracellular diffusion. Recent applications of these modelling frameworks to microbial communities are reviewed in [22].

The choice of modelling framework is influenced by the system under consideration, the modelling objective, the data available, and the computational resources at hand. For each modelling framework, there are numerous open-source simulation packages available. The choice of software depends primarily on whether the built-in features are suitable for the application at hand. Reusability can be a challenge due to the diversity of programming languages and documentation formats employed. Some groups are developing packages with graphical user interfaces to facilitate reuse of their simulation software [23–25].

Predictive models are necessary for applications that require precise design and manipulation of complex microbial communities [8]. These applications include human and animal health, food production, and environmental remediation. ABM and PDE models are suitable for modelling microbial growth in heterogenous environments such as the mammalian gut and soil. To make accurate predictions, these models must be validated against experimental data, such as direct observations of populations of cells growing in spatially distributed environments. Observations at single-cell resolution can simultaneously provide data at the single-cell, population, and community scale. Such single-cell data are especially valuable for validating ABMs that aim to capture emergent population-level features by modelling single-cell behaviour [26,27]. Validation against independent patterns occurring at multiple scales generally improves model accuracy and predictive power [28].

To define the scope of the following discussion, we first establish a working definition of “microbial community.” Although a broad definition could be “microbes living together,” there is no consensus on how much variability is required to distinguish a microbial community from a microbial “monoculture”: Even these exhibit some degree of genetic and phenotypic variability. For this review, we define the fundamental property of a community as the presence of at least 2 distinct characterized organism types, and thus we exclude monocultures that have developed some uncharacterized genetic heterogeneity. For details on the application of single-cell technologies to investigations of heterogeneity in such monocultures, the reader is referred to [29,30]. A number of distinct categories of communities have been investigated at the single-cell level [31]:

- **Communities of “isogenic mutants”** are cocultures composed of at least 2 strains derived from the same parent that exhibit some genetic differences (due to either engineered or natural genetic alterations). Communities of isogenic mutants commonly serve as testing grounds for design and characterization of ecological interactions.
- **Designer laboratory communities** are composed of distinct species purposefully combined in a laboratory environment. The number of species is typically small compared to natural communities.
- **Natural communities** are sampled from natural or engineered environments (e.g., soil, animal guts, wastewater treatment plants, fermentation cultures).

Below, we provide a brief overview of current techniques for collecting single-cell level observations of microbial communities. We then survey how such measurements have been used to calibrate computational models of community dynamics, and we highlight systemic approaches that could be used to improve the rigour of model calibration procedures. Finally, we discuss techniques from (macrobial) ecology, topology, and data science that hold promise for facilitating efficient calibration.

## 2. Single-cell level observations of microbial communities

**Fig 2:**
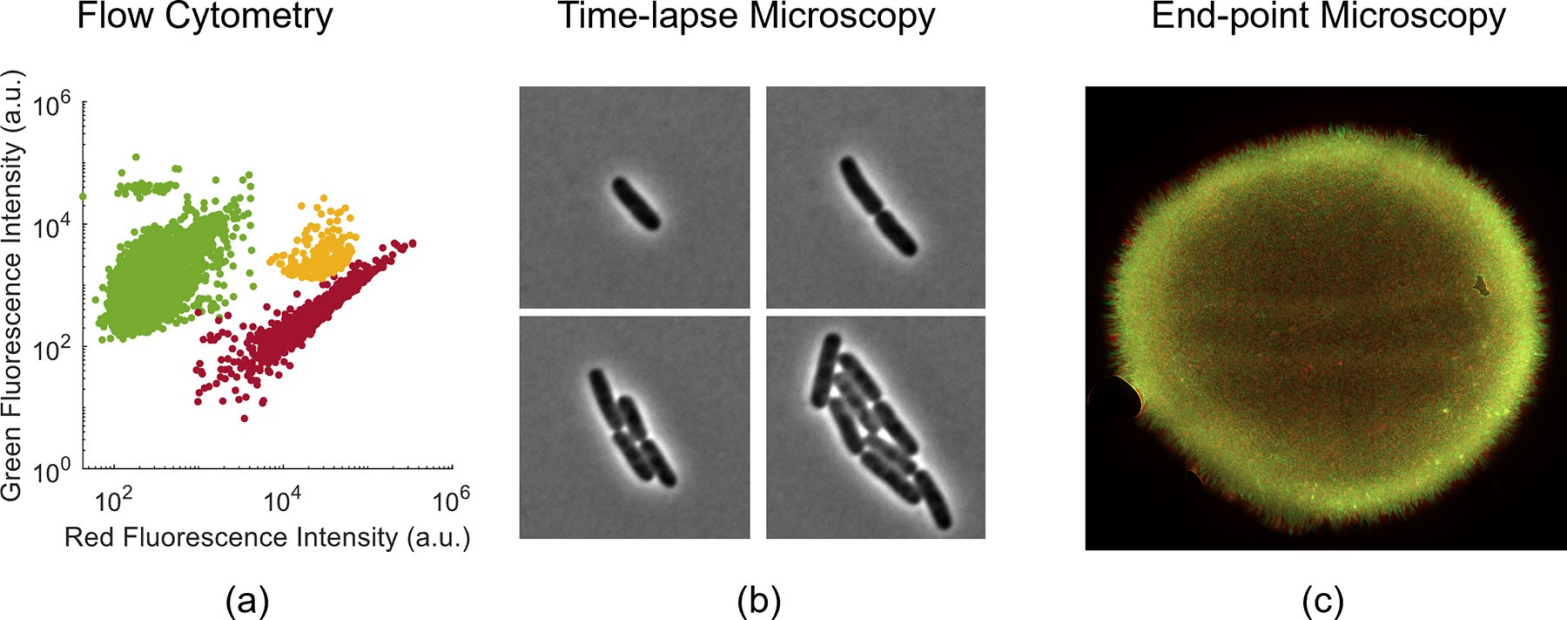
Single-cell measurement techniques for microbial communities. (a) Flow cytometers can measure cell fluorescence and morphology in large sample sizes. (b) Time-lapse microscopy allows for direct visualization of physical cell–cell interactions and quantitative measurement of single-cell characteristics over time. (c) End-point microscopy can illustrate large-scale spatial patterns and features of cell arrangements in 2 or 3 dimensions.

Microbial communities are most commonly observed by flow cytometry and microscopy (Fig 2). Flow cytometry can be used to categorize cells by their morphology and physiological characteristics (e.g., fluorescence). Modern flow cytometers can process approximately 10^4^ cells per second. The resulting large samples can provide robust statistics characterizing heterogenous populations. The availability of several out-of-the-box software packages for flow cytometry [32,33] makes processing cytometry data straightforward in comparison to microscopy images, which usually call for customized image processing routines [34].

Flow cytometry is often used to determine the relative fraction of subpopulations within a community. Cells are distinguished by, e.g., fluorescent labeling, viability staining, or morphological differences. This approach has been used to measure short-term population dynamics in synthetic consortia [35], rates of plasmid propagation [36,37] and monitoring of eco-evolutionary feedback between cooperators and cheaters [38], among others.

Time-lapse microscopy has been used to collect spatiotemporally resolved measurements of microbes in confined environments. Cells are typically observed growing in monolayer using a widefield microscope, but multilayer growth can be resolved by confocal microscopy [39–41]. Fluorescence microscopy experiments can generate data on the spatiotemporal positions of cells and their fluorescence-associated phenotypes. Time-lapse images can be processed to reveal growth rates, lineages, gene expression levels, and to infer intercellular interactions, which together give rise to spatiotemporal features at the population level.

Time-lapse experiments generally involve observation of cells growing under agar pads or within microfluidic devices. Agar pads are simple to use but suffer the limitations of batch cultures such as transient effects of nutrient depletion, desiccation, waste product accumulation, and crowding [42,43]. In contrast, microfluidic devices offer more controlled and sustained environments, in which multiple cell generations can be observed through transient and steady-state growth conditions [44].

Quantitative analysis of time-lapse microscopy images demands the use of cell segmentation and cell tracking algorithms [45,46]. Analysis of time-lapse images reveals individual cell properties such as elongation rate, motion, and lineage, as well as population-level features such as population density and species abundance. Populations can sometimes be discriminated by morphology, but it is more common to use fluorescent markers. Moreover, fluorescence intensity can be used as a readout of an internal genetic state [47]. Time-lapse microscopy has been used to obtain both single-cell and population measurements in both isogenic mutant [48–54] and laboratory designer communities [55,56].

By correlating individual cell elongation rates with counts of neighbouring cells, researchers have gained insight into cell–cell interactions such as metabolite exchange [57,58] and antibiotic efflux [59]. Such studies can take advantage of microfluidic device designs that constrain the proximity of neighbouring cells. For example, Moffitt and colleagues [60] and Gupta and colleagues [61] designed microfluidic devices permitting nutrient exchange between 2 physically separated populations.

Contact-dependent interactions can be inferred by comparing changes in cell state to the presence of directly neighbouring cells. This approach has been employed by several groups studying type VI secretion (toxin delivery) systems (T6SS). LeRoux and colleagues [62] and Smith and colleagues [63] measured the efficiency of target cell lysis as a function of contacts made. Steinbach and colleagues [64] investigated how the accumulation of dead cell debris reduces T6SS killing efficiency. Time-lapse microscopy studies of conjugation (contact-dependent horizontal gene transfer) have demonstrated the influence of contact mechanics on conjugation frequencies [65] and have also revealed enhanced gene transfer by transformation (uptake of DNA from the environment) in predator–prey communities [66,67].

Some investigations of community behaviour have relied on representative snapshots of spatial structure provided by single time point (i.e., end-point) microscopy. This approach is useful when time-lapse approaches may not be feasible, such as in highly structured environments like biofilms and solid matrices [68], or in microdroplets [69]. (The recent time-lapse work of Hartmann and colleagues [70] and Nijjer and colleagues [71] characterizing biofilm growth is a notable exception and may represent a new paradigm for such measurements.) Data on 3D arrangements within communities provide quantitative insights on how spatial distributions impact phenotype [72] and vice versa [73,74]. Co-occurrence networks in nonspatial environments can also be determined in microdroplets [10,69]. End-point cell arrangements constrained in 2 dimensions have been used to measure interaction ranges of quorum sensing mechanisms involved in horizontal gene transfer [75].

## 3. Calibration of spatial mathematical models of microbial communities against single-cell measurements

When mathematical models are employed to explore a range of possible behaviours, parameterizations need not accurately capture specific observations (e.g., [76–78]). In contrast, when models are used for predictive purposes (as in most engineering applications), models must be fit to observations. In such cases, descriptions of the formulation, calibration, and validation of the model are needed to specify the predictive strengths and limitations of the model. A first step in communicating a model’s formulation is the statement of the model’s purpose, which clarifies the scope of the model structure and parameterization. This is highlighted in the ODD (overview, design concepts, and details) protocol [79,80], a formal framework for documenting ABMs. The protocol has been used in documenting several spatiotemporal ABMs of microbial communities [25,81–83]. In macrobial ecology, this protocol is often used as part of a larger modelling methodology called pattern-oriented modelling [84,85], discussed further in Section 4.1.

Model calibration is the process of assigning values to model parameters to best reproduce available data. Ideally, calibration is complemented by uncertainty analysis, which gauges the degree of confidence in model predictions and parameter estimates through, for example, identifiability analysis and sensitivity analysis [86,87]. The simplest approach to model calibration is to characterize each component of a system independently. This approach is suitable for simple processes, such as growth or diffusion, for which direct measurements can be made.

In contrast, it is often the case that calibration of biological models must be posed as an *inverse problem*: Properties of system components cannot be measured directly and must instead be inferred from observations of overall system behavior. In such cases, model calibration involves selection of a “goodness of fit” function, typically defined as a sum of squared errors (SSE)—the SSE measure aligns with a maximum likelihood measure under idealized assumptions about system and noise structure [88]. When calibrating linear models, a rich theory provides robust uncertainty analysis, such as 95% confidence intervals for parameter estimates and model predictions. When addressing nonlinear dynamic models, the theory provides less support; models that minimize the SSE can be found only through nonlinear optimization procedures (typically iterative global optimization routines; [89]), and uncertainty analysis is approximate (though there are uncertainty tools designed for nonlinear systems, e.g., profile likelihoods; [90]). Bayesian calibration methods, such as approximate Bayesian computing [91], offer an alternative to global optimization searches. Bayesian methods refine uncertainty distributions for model parameter values by comparing with experimental observations.

For nonspatial models (e.g., ODEs), systematic model calibration approaches have become standard in the field of computational biology, as reviewed in [86,87]. Such ODE models are used to describe microbial community dynamics through compartmentalization. For example, Gupta and colleagues [61] used a compartmental ODE model to investigate the behaviour of physically separated microbial populations; they calibrated their model parameters using a standard SSE-minimizing approach.

### 3.1 A survey of calibration approaches for spatiotemporal models

**Fig 3:**
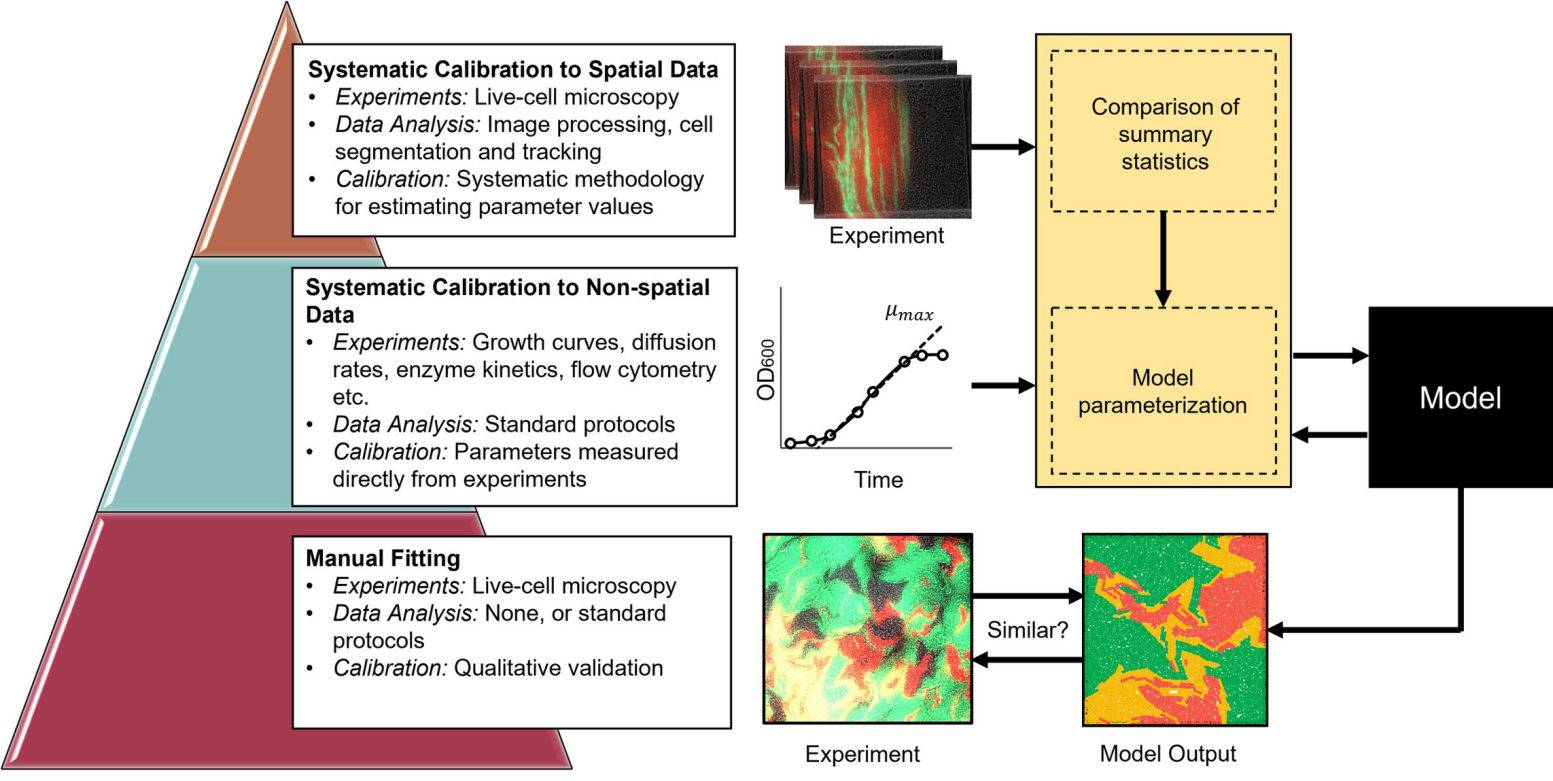
Model calibration techniques for spatiotemporal models of microbial communities. **Manual fitting** involves direct adjustment of parameter values to achieve qualitative agreement between model predictions and observations. **Nonspatial calibration** is often systematic (based on a goodness of fit function) but is based on experiments that do not incorporate the spatial features of the system. **Spatial calibration**, against spatially distributed data, can be systematic (SSE-based) but must rely on summary statistics collected from the data.

In this section, we survey strategies recently employed for calibration of spatiotemporal models of bacterial communities against observations at (or near) the single-cell level. The corresponding data (as described in Section 2) are complex, and calibration of these models is challenging. Calibration strategies used in recent publications can be roughly classified into 3 categories (Fig 3): manual fitting, systematic calibration to nonspatial data, and systematic calibration against spatial summary statistics.

The simplest calibration approach is **manual fitting**, by which model simulations are qualitatively compared to observed data, and model parameter values are adjusted to arrive at a satisfactory alignment. This approach, often used to extend previously established model structure, is exemplified by comments such as “We chose model parameters to qualitatively fit the experimental results…” [51], and “We adjusted the parameters of our simulations until the behaviour matched the images of real cells…” [92]. Manual fitting is a pragmatic means to arrive at qualitative agreements between model and data, which is often perfectly appropriate for modelling objectives. However, it is poorly suited for situations in which precise calibration is required, it is unlikely to provide a robust search of high-dimensional model spaces, and it is unsatisfactory in terms of reproducibility.

A second common calibration tactic is to apply **systematic calibration to nonspatial data** compared with an aggregate model output. For example, in their study on the inhibitory role of antibiotic efflux activity from neighbouring cells, Wen and colleagues [59] observed interactions between 2 bacterial populations, one of which expressed antibiotic efflux pumps. They used an SSE-based approach to estimate growth and inhibition parameters from data obtained by suspension growth experiments. They then used those parameters in an ABM that supplemented findings from additional single-cell experiments. Another example is provided by the work of Pande and colleagues [93], who investigated the role of spatial segregation in cross-feeding populations. They used in-suspension growth curves obtained over a range of nutrient concentrations (via Monod growth kinetics) to fit growth parameters, which were then applied to a spatial ABM of the consortium. Such strategies rely on an assumption that the behaviours measured in suspension are representative of behaviour in the spatially structured environments under investigation.

Finally, in some instances, model developers have made full use of spatiotemporal data by **systematic calibration against spatial summary statistics** that capture the spatiotemporal aspects of primary interest, fitted with an SSE-based protocol. For example, Hartmann and colleagues [70] validated a 3D cell tracking algorithm and calibrated an ABM by minimizing the error between measured and simulated summary statistics in a growing biofilm. Another example is provided by Leaman and colleagues [94], who collected summary statistics from spatial distributions of cells and then used a global optimization scheme to fit parameters of an ABM. Such systematic calibration approaches can be resource-intensive, often requiring detailed image processing pipelines (e.g., [70]), or numerous auxiliary experiments to fit physical or chemical parameters. For example, Leaman and colleagues [94] needed to measure both diffusivity of solute particles in the presence of bacteria and activation time for gene expression controlled by a quorum sensing molecule before calibrating the rest of the model’s parameter values.

In the next section, we present a collection of summary statistics that are suitable for validation of spatiotemporal models of bacterial community dynamics. Use of these summary statistics typically requires development of a data processing pipeline for image processing and summary statistic calculation.

### 3.2 A catalogue of spatiotemporal summary statistics for microbial community dynamics

As discussed in Section 2, modelling projects are frequently built on spatiotemporal data that are rich and complex, resulting in a tendency to aim for qualitative agreement or calibration against nonspatial observations. Hartmann and colleagues [70] and Leaman and colleagues [94] provide examples that make more complete use of the richness of spatiotemporal data by selecting summary statistics to capture key spatial features in a quantitative manner and applying SSE-based calibration to ensure accurate representation. As we discuss below in Section 4.1, this strategy has been adopted for many modelling projects in the macrobial ecology community, where spatiotemporal datasets of this type have been collected for decades. One of the challenges of this approach is identification of appropriate summary statistics. These should (i) capture relevant features of the system’s behaviour; (ii) be represented by model outputs; and (iii) be computationally tractable (in terms of image processing). In this section, we survey summary statistics that have been used to capture spatiotemporal features of microbial dynamics (Table 1), along with some examples from macrobial ecology that hold promise for use in this context.

**Table 1:** Summary statistics for quantifying features in microbial communities.

Statistic	Goal of Statistic	Computational Details	Reference
**Summary Statistics for Single-Species Populations**			
*Microcolony shape*			
Microcolony aspect ratio	Quantify eccentricity of developing colony	Standard image processing feature, defined in 2D (or 3D, projected to 2D)	[70,95]
Dyad structure	Characterize structure of 2-cell “colony” immediately before the second division	Normalized dot product of the 2 cells’ orientation	[95]
Biofilm base circularity	Characterize shape of the biofilm base	Unity minus aspect ratio of projection onto the horizontal plane	[70]
*Internal microcolony structure*			
Microcolony density	Quantify packedness of cells within developing colony	Standard image processing feature, defined locally or globally	[70,95–97]
Order parameter	Quantify anisotropy within developing colony	Mean of projections of orientation of neighbouring cells; defined per-cell, recorded as a colony average or as a distribution	[70,95–99]
Correlation length of scalar order parameter	Characterize “patchiness”: spatial scale over which orientation of neighbouring cells is aligned	Correlation of orientation as a function of distance; can be compared as a mean or a distribution	[98]
Micropatch area	Quantify “patchiness”; similar to correlation length of scalar order parameter	Cells are clustered into patches based on contact and relative orientation	[100]
Topological defect density	Characterize “patchiness”: density of topological defects (i.e., discontinuities in the order-parameter field)	Algorithm provided in [101]	[98]
Defect velocity	Characterizes the evolution of a microcolony’s internal structure	The position of topological defects is tracked over time	[98,99]
Age distribution of cell poles within the developing microcolony	Characterize degree of mixing during colony development	Simple measure is distance from centre of colony to oldest cell poles. More complete measures additionally account for younger poles	[102]
*Other metrics*			
Orientation of cells at the microcolony boundary	Characterize tendency of boundary cells to align with the colony boundary	Colony boundary must be determined by a smoothing operation; cells on the boundary and the corresponding boundary orientation must be identified	[99]
Gradient of cell velocity normal to microcolony boundary	Characterize growth inhibition due to pressure gradients	Measured by particle-image velocimetry	[96,97]
Cell–cell distance	Characterize cell spacing	Centroid-to-centroid distance to nearest neighbour	[70]
Vertical and radial alignment	Characterize 3D structure; identify transition from monolayer to multilayer growth	Angle formed by the z-axis and cell’s major axis	[70]
**Summary Statistics for Multispecies Populations**			
*Composition*			
Single-strain population counts; population fractions	Captures population abundances	Cell counts for each population	[49,58,63,103,104]
Shannon species diversity index	Species biodiversity metric	Determined from cell counts for each population	[105]
*Spatial configuration; nearest cell–cell adjacencies*			
Shannon entropy	Quantify randomness of pixel identities in an image	Total population of each species, heterospecific neighbouring cell counts	[35,105]
Intensity correlation quotient	Characterize colocalization or exclusion of pairs species in space	Determined by sum of pixel intensities for each fluorescence channel	[35]
Contagion index	Quantify dispersion and intermixing of different populations; deviation from maximum entropy state	Total population of each species, heterospecific neighbouring cell counts	[106]
Probability matrix for adjacent species identities	Global quantification for likelihood of nearest interspecific adjacencies	Identify neighbour to cell centroid	[73]
Neighbour index	Characterize interspecific adjacencies relative to the initial adjacencies	Count physical contacts between pairs of cells of different phenotypes	[104]
*Spatial configuration; cell–cell adjacencies within a neighbourhood*			
Proportion of conspecific neighbours	Quantify interspecies mixing from a probability distribution; *β*-diversity metric	Probability that a cell is located a defined distance away from other members of its own species	[107,108]
Structure factor	Quantifies characteristic length scales of spatial patterning; used to characterize transition from well-mixed to structured populations	Normalized spatial Fourier transform of image data	[109]
Segregation index	Normalized metric to quantify population segregation	Cell neighbourhood interaction distance, heterospecific neighbouring cell counts	[110–113]
*Spatial configuration*: *shape*			
Colony edge roughness	Increased in communities with antagonistic interactions	Standard deviation of microcolony radius	[103]
Fractal dimension	“Jaggedness” of species boundaries	Distance of each pixel to nearest border of 2 different populations; algorithm provided in [114]	[115,116]
*Spatial configuration; sectors*			
Intermixing index	Estimate spatial mixing between multiple species	Average number of species transitions along a straight line or arc	[117,118]
Single-strain sector size	Indirect measurement of spatial mixing between multiple species	Length scale of single-strain patches	[104,119]
*Temporal*			
Order parameter; phase transitions	Quantify synchrony in gene expression between populations	Spatiotemporal data are processed into kymographs; algorithm introduced in [120]	[53]

Monolayer growth is the simplest setup for observing single-cell characteristics of microbial population dynamics. In this setting, single-cell features such as elongation rate and division length threshold can be measured directly. The simplest population to study is an isolated microcolony descended from a single cell. Several groups have proposed summary statistics to capture development of such microcolonies. Volfson and colleagues [96] were one of the first to compare simulations of an ABM to time-lapse images of developing microcolonies within microfluidic devices. They calculated **microcolony density**, a **cell velocity gradient**, and an **order parameter** quantifying the global anisotropy in cell orientation. These summary statistics have been used to calibrate parameters governing physical interactions between rod-shaped bacteria in more recent ABM projects (e.g., [97]). Doumic and colleagues [95] used similar metrics in their model of microcolony growth that incorporates unequal mass distribution upon cell division. They also considered the microcolony aspect ratio and the relative orientation of the 2 daughter cells just prior to the second division (called “*d*_*2*_” in Doumic and colleagues, and **dyad structure** in Table 1). Doumic and colleagues highlight additional summary statistics for microcolony development: **orientation of cells at the colony boundary** (this is referred to as “active anchoring” in [99]) and relative position with respect to **age of individual cell poles** within the colony [102].

Monoculture microcolony development has also been characterized using summary statistics from liquid crystal theory. These measures quantify the degree of physical alignment between neighbouring cells. The order parameter used by Volfson and colleagues [96] (mentioned above) is one such example. van Holthe tot Echten and colleagues [98] took the mean of this measure over the entire colony to compare the evolution of real microcolonies to ABM simulations. Dell’Arciprete and colleagues [99] use this same statistic, which they call the global order parameter, to summarize overall microcolony orientational disorder. Orientational order can also be quantified with discrete measurements. You and colleagues [100] investigate the inner structure of microcolonies by segmenting them into patches of similarly oriented cells and comparing the distribution of **patch areas** between ABM simulations and experiments. van Holthe tot Echten and colleagues [98] also use **correlation length of the scalar order parameter** (a measure of patch size) and **topological defect density** as additional measures of microcolony structure. (Topological defects are discontinuities in the orientation field that arise at boundaries between patches of similarly oriented cells. These are locations that lack a representative trend in orientation.)

Studies of monolayer growth provide valuable insights into microbial activity, but they represent an idealized version of microcolony formation. In contrast, Hartmann and colleagues [70] present a comprehensive study of biofilm formation in 3 dimensions. They present novel imaging and image-processing tools that allow single-cell level tracking of a *V*. *cholera* biofilm from a single progenitor to about 10,000 cells. To quantify growth of this population, they make use of a collection of spatial summary statistics: **vertical and radial alignment**, local order, **cell-to-cell distance**, **density**, and **aspect ratio** of overall population and biofilm base.

**Fig 4:**
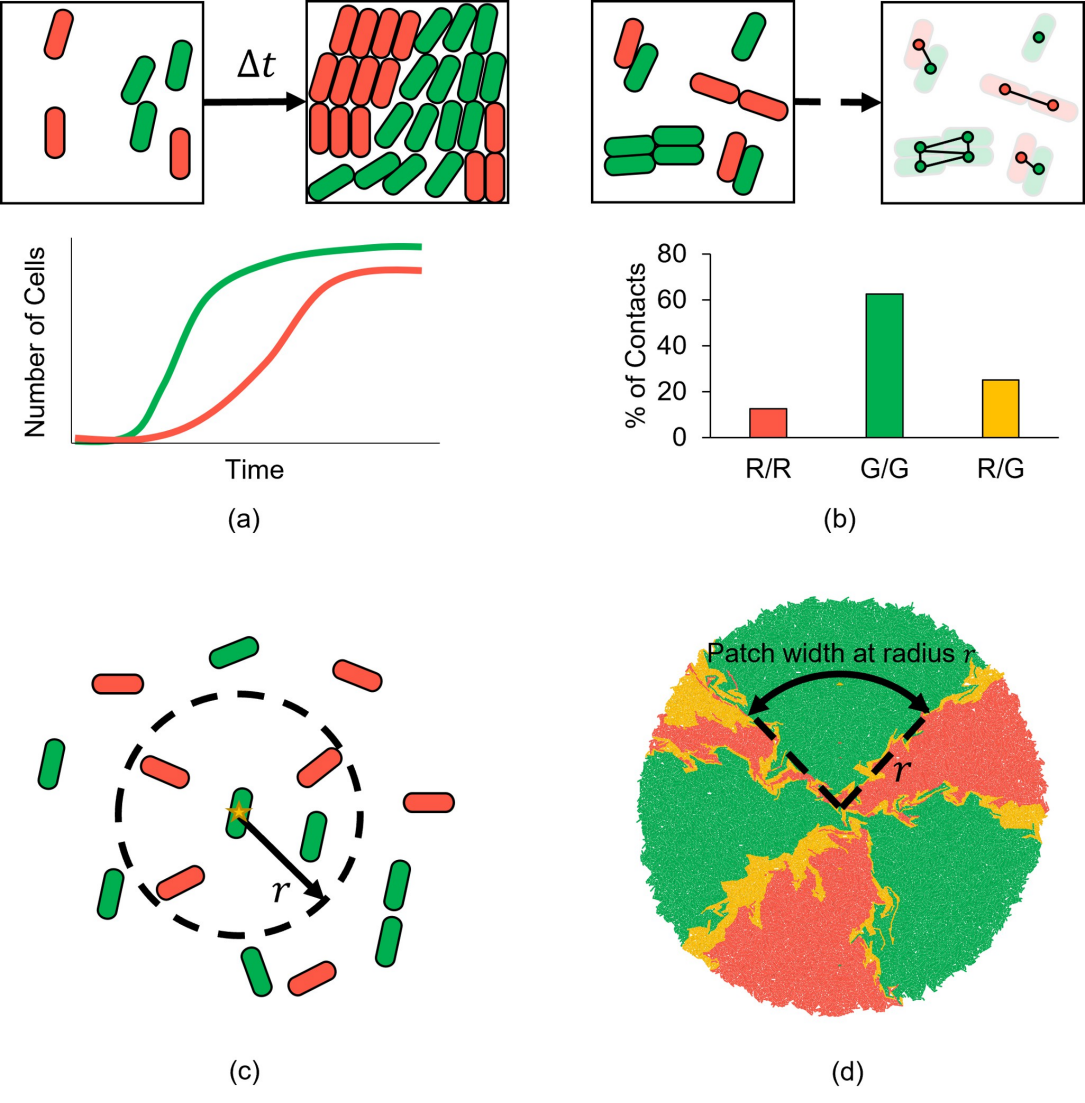
Calculating spatiotemporal summary statistics for microbial communities. (a) Population counts over time capture the overall dynamics in a multispecies community. (b) The frequency of adjacent species in physical contact, determined by a contact network, provides a measure of intermixing between different species. (c) Summary statistics can be calculated from data averaged within a particular cell’s neighbourhood. (d) Single-species patch metrics, such as patch width and number of sectors, are useful for quantifying spatial patterns on a larger colony scale.

In multispecies communities, **population counts of each species** are a simple, key summary statistic that encapsules population dynamics (Fig 4A). Microbial interactions that have been summarized using population fractions include secretion of nutrients and toxins [103], cell lysis by T6SS [63], and competition for space in microfluidic traps [49]. While population fractions are often measured globally, localized measures are also used. For example, Bottery and colleagues [104] measured population fractions as a function of a microcolony radius, while Dal Co and colleagues [58] measured population fractions within a given radius for each cell to quantify the length scale of interactions mediated by secretion of diffusible molecules. In microbial communities consisting of a large number of species, an index summarizing biodiversity, such as the Shannon diversity index [105], may be more informative than specific population fractions.

Most summary statistics that describe spatial patterns in multispecies communities quantify the degree of interspecies mixing or (conversely) of monospecies patchiness. These measures are commonly used in the field of landscape ecology [121]. Metrics from landscape ecology traditionally rely on counting adjacencies between image pixels, each of which is assigned a value corresponding to its dominant occupant. These metrics can be applied in the same manner for low-magnification microscopy images of microbial colonies. **Shannon entropy** is a canonical metric for species mixing [105]; it quantifies the overall disorder between any number of populations by counting like- and non-alike pixel adjacencies. Kong and colleagues [35] used this measure to assess the extent of red-green pixel colocalization in 2-strain microbial communities from microscopy images taken at 7× magnification. Li and Reynolds [106] developed a **contagion index** [122] that quantifies the deviation from the maximum entropy state using the same type of pixel adjacency counts. This contagion index is used widely in landscape ecology because it captures both aggregation of single populations and intermixing of different populations.

Landscape ecology metrics could be extended to higher-magnification single-cell data by generating a physical contact network and accounting for nonrectangular adjacency structure (Fig 4B). Alternatively, single-cell images can be smoothed until continuous single-species patches are formed [104,119]. Other metrics defining patch shape, aggregation, and species/strain diversity (discussed below) could also translate to the single-cell level. Mony and colleagues [123] discuss applications of other higher-level principles from landscape ecology to analysis of microbial community assembly and structure.

While the contagion index has not yet been applied to microbial studies, related measures have been used. For example, Bottery and colleagues [104] counted physical contacts in pairs of cells of differing strain/species (Fig 4B). They normalized these counts to initial neighbour counts, arriving at a metric they called the **neighbour index**. An alternative intermixing measure that does not require counting all physical contacts between cells is a **probability matrix for adjacent species identities**, computed by identifying the species/strain of a cell’s nearest neighbour. Glass and Riedel-Kruse [73] used this type of measurement to quantify effects of surface nanobodies and antigens on cell–cell adhesion.

Summary statistics that describe proportions of species within some defined neighbourhood (Fig 4C) are also used to describe intermixing of microbial populations. The **segregation index** [110–113] measures the degree to which cells within a given neighbourhood radius are related to one another (by genotype or phenotype). In this case, the radius is defined as the distance over which interactions mediated by small molecules are expected to equally influence all cells within the neighbourhood [113]. The segregation index has been applied to simulated data in numerous microbial ABM studies but has yet to see use in the context of single-cell microscopy data. Generalizing this measure, the **proportion of conspecific neighbours** is defined as the probability that 2 randomly selected individuals separated by some defined distance will belong to the same population [107]. Computing this metric over a large sample of individual pairs provides the proportion of conspecific neighbours as a function of distance. An alternative way to define a neighbourhood is by a characteristic length scale. McNally and colleagues [109] used a **static structure factor** to identify transitions from well-mixed to segregated states in antagonistic 2-strain communities. This metric was computed using Fourier transforms of binarized pixel intensities to assess spatial (patch size) frequencies of each strain within a characteristic length scale.

Other metrics for defining intermixing on larger scales use the number of single-species patches as a measure of interspecific mixing. For example, the **intermixing index** is determined by the average number of single-species patch transitions along a line or arc. This metric has been used as a measure of species colocalization in low-magnification images of biofilms and microbial colonies [117,118]. Blanchard and Lu [103] and Bottery and colleagues [104] used the **number of single-strain sectors** of a circular colony to characterize spatial patterns in 2-strain communities growing on a surface with open boundary conditions (Fig 4D).

Some spatial patterns may be observable from the physical shape of single-species sectors or colony boundaries (Fig 4D). Kan and colleagues [115] and Rudge and colleagues [116] measured the **fractal dimension** of species patch boundaries, which quantifies jaggedness. Blanchard and Lu [103] noted that the **roughness of a growing colony’s edge** increases when there are antagonistic interactions between different strains. Amor and colleagues [119] and Bottery and colleagues [104] used **sector widths** as an indirect measurement of spatial mixing, because larger widths imply less mixing. The perimeter-to-area ratio of single-species sectors could also be appropriate as a summary statistic for shape [121], although it has not been used yet in microbial studies.

The summary statistics described above are applicable to end-point measurements. Of course, these can be measured through times series, but alternative measures rely explicitly on time series, e.g., through windowed averages and autocorrelation [124]. Periodicity can also be used as a temporal metric, quantified by, e.g., a **periodic order parameter**, as demonstrated by Kim and colleagues [53], who summarized spatiotemporal synchronization of gene expression in a 2-strain community. Time derivatives of summary statistics can also be assessed. For example, Dell’Arciprete and colleagues [99] and van Holthe tot Echten and colleagues [98], discussed above, both use the **velocity of topological defects** to characterize microcolony dynamics.

## 4. Outlooks

There is no doubt that spatiotemporal models of microbial communities will continue to grow in complexity (and corresponding computational requirements) as researchers continue making advances in synthetic ecology, in microbiome engineering, and in characterizing natural systems. In this section, we survey some outlooks for standardizing and streamlining model development and validation.

### 4.1 Pattern-oriented modelling as a guideline for standardizing microbiological models

It can be challenging to describe ABMs efficiently, but complete descriptions are crucial; incomplete reporting leads to difficulties with subsequent implementation and replication, as demonstrated by Donkin and colleagues [125] and discussed in [124,126]. Furthermore, systematic model documentation can improve model quality by enforcing critical thinking about the model’s objective, formulation, implementation, and validation. In surveying modelling practices for microbial communities, we found that modelling and documentation practices vary considerably, especially regarding model calibration. A systematic framework for model development and testing, referred to as pattern-oriented modelling (POM) [84,85], sees frequent use in macrobial ecology and has been used occasionally in microbial settings as well [127–130].

POM addresses “the multi-criteria design, selection and calibration of models of complex systems” [85]. The framework formalizes all stages of the modelling pipeline, from model formulation, to testing, to calibration and validation. The “patterns” in POM are any quantifiable features of model simulations; we referred to these as summary statistics in Section 3. These measures are most useful when they span ecological scales: individual, population, community, ecosystem. As highlighted by the POM framework, summary statistics facilitate validation by reducing system dimensionality [131]. Moreover, they can guide model formulation by focusing attention on the aspects of simulations that will be quantitatively captured.

POM’s model validation strategy is standard [124,131]: begin with qualitative comparison of model predictions with experimental data, then sample the parameter space to determine the sensitivity of summary statistics to parameter values (typically done in a one-at-a-time fashion, given computational costs) (e.g., [108]). Acceptable parameter fits are then determined based on systematic minimization of SSE quality-of-fit measures using a weighted average of the summary statistics, as in, e.g., [132–134]. Documentation of all model formulations and parameter sets tested can provide insights into model behaviour and can potentially reveal underlying mechanisms of emergent community properties.

### 4.2 Feature identification through topological data analysis

The selection of appropriate “patterns” is a subjective task, as acknowledged by the architects of POM [85]. Moreover, it is not always clear how best to quantify these patterns as summary statistics once they have been identified. Some features are easy to represent numerically (e.g., average population density), but many relevant patterns are qualitative, or manifest as complex spatiotemporal configurations. In some cases, existing theory can offer tools to quantify these features, such as order parameters from liquid crystals, or Fourier coefficients to identify feature scales (both described in Section 3). In the absence of such tools, many researchers rely on visual inspection, which introduces subjectivity into the calibration pipeline. A generic approach to pattern identification is provided by the recently developed tools of topological data analysis (TDA).

TDA provides tools to quantify topological (i.e., qualitative) features within datasets. It can be applied either to discrete datasets, like those from ABMs, or to continuous data, like those from PDE models. TDA encompasses a wide variety of tools and techniques. Here, we focus on the most popular: persistence homology. (For a general overview of the field, see [135]; a broad discussion of applications to biology is presented in [136].) Persistence homology can be thought of as a nonlinear analogue of a more familiar technique: principle component analysis (PCA) [137]. PCA is used to identify the variational structure within datasets: If there are correlations within the data, the points will tend to cluster around certain linear subspaces (lines, planes, etc.) and the data will exhibit less variation in the directions perpendicular to these subspaces. One application of PCA is dimensionality reduction. Data can be projected onto these linear subspaces, thereby reducing the dimensionality of the dataset with minimal information loss.

**Fig 5:**
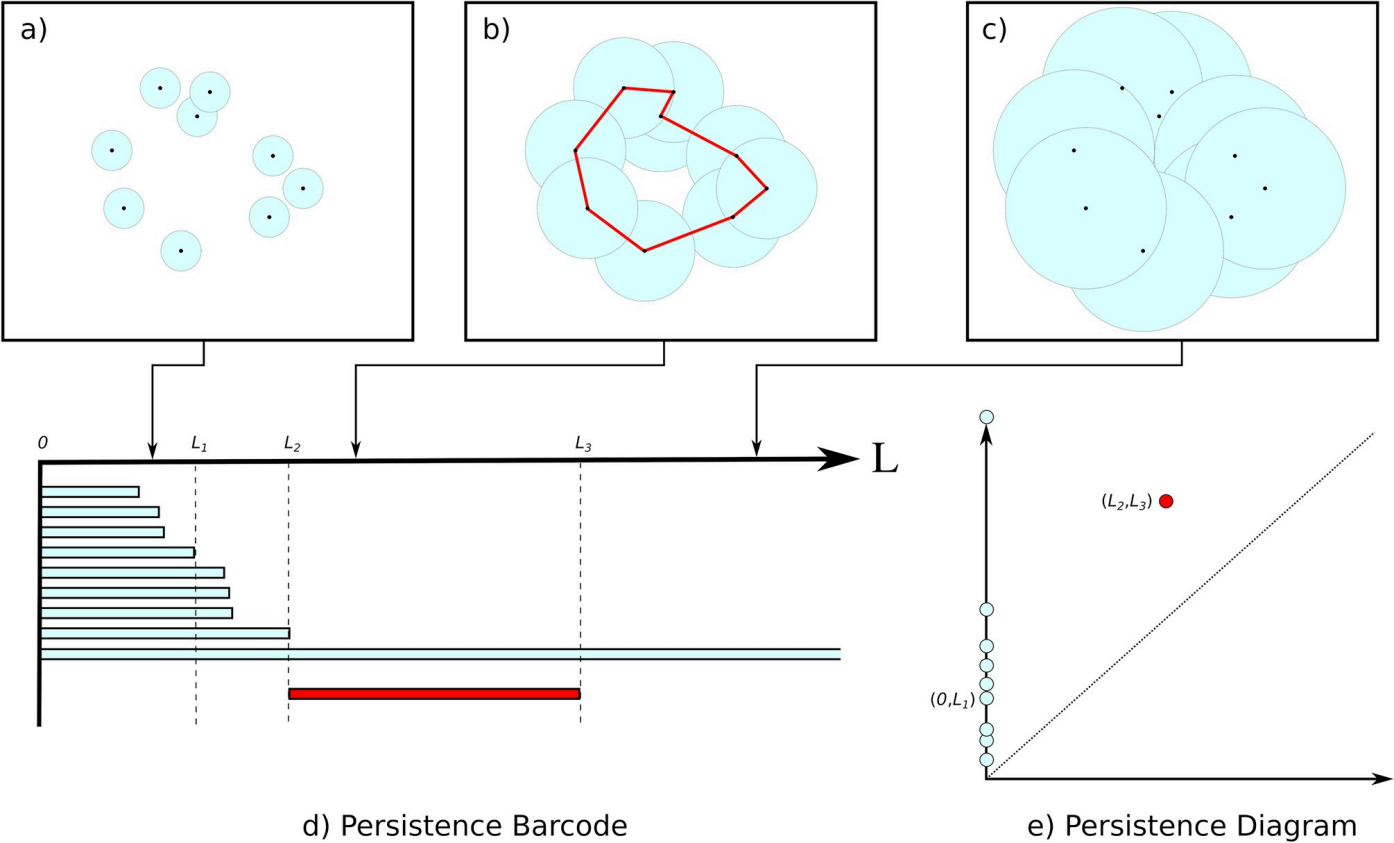
Persistence homology. Panels (a-c) illustrate the topology of a dataset changing as the length scale, *L*, is varied. (a) For small values of *L*, the balls (disks) are mostly disconnected; only 2 of the 9 intersect. (b) At an intermediate scale, all 9 balls intersect, forming a single connected component, giving rise to a loop. (c) At larger scales, there is a single connected component and no loop. (d) The progression illustrated in (a-c) is documented in the persistence barcode; the blue bars correspond to separate connected components, the ends of which corresponds to intersection (merge) events, e.g., at *L = L*_*1*_. The red bar corresponds to the loop, which forms at *L = L*_*2*_ and which becomes filled in at *L = L*_*3*_. (e) The same information can be represented in persistence diagram in which the (*x*,*y*) coordinates of points correspond to the right and left ends, respectively, of each bar in the barcode.

Whereas PCA identifies linear structure within a dataset, persistence homology identifies arbitrary nonlinear structure. A basic persistence homology workflow for discrete data can be described as follows (Fig 5). First, data are represented as a collection of points in some (typically) high-dimensional space that characterizes features of interest. If, for example, TDA were to be used to characterize the results of an agent-based simulation, each point might represent an individual agent, with the coordinates corresponding to features of that agent: e.g., species, position, length, orientation. To proceed, a length scale *L* is chosen, a ball of radius *L* is constructed around each point, and the topological features of the shape thus produced are determined, e.g., connected components and loops. This analysis is repeated over a wide range of length scales (Fig 5A–5C). At small scales, it will produce only a cloud of disconnected points. As the length scale, *L*, increases, neighbouring balls intersect, forming larger and larger structures until, finally, they merge into one fully connected component. As *L* varies, the length scales at which various topological features occur (i.e., over which they persist) is recorded. Each topological feature is thus associated to a pair of numbers: the smallest and largest scales at which the feature exists. These can be represented by a persistence “barcode” in which bars are plotted against length scale. Each bar corresponds to topological feature; the bars represent the length scales over which the features occur. (In Fig 5D, the teal bars end at scales at which connected components merge.) As an alternative visualization, the pairs of length scales associated to each topological feature can be plotted in a persistence diagram (Fig 5E; any features near the diagonal occur over only a short range of length scales and may be dismissed as spurious). A similar analysis can be applied to continuous data (e.g., from a PDE model) by characterizing the topology of the sublevel sets of a continuous function, such as population density [135].

Much like PCA, persistence homology provides a natural way to reduce the dimensionality of the dataset. Features that persist over a narrow range of length scales are likely spurious and can be discarded. The features that persist over a wide range of length scales more likely correspond to meaningful structure in the dataset: Connected components might correspond to discrete clusters, loops to periodicity. Persistence diagrams can be compared to one another using a variety of metrics, allowing them to be used directly for model calibration. They can also serve as a starting point for development of custom summary statistics.

This type of analysis was applied by Topaz and colleagues [138] to study ABMs demonstrating swarming behaviour. In that case, the agents each have a position and a velocity, so clusters of points in position-velocity space correspond to swarms—closely grouped agents exhibiting collective motion. Previously, researchers had developed case-specific order parameters to quantify such behaviour [139–141]. By applying TDA, Topaz and colleagues [138] were able to detect features that the previous ad hoc metrics failed to quantify. Similar approaches could be applied to characterize the dynamics of microbial communities.

### 4.3 Machine learning algorithms to accelerate model calibration

Conducting global parameter sweeps over high-dimensional parameter spaces is often infeasible due to computational limitations. For spatiotemporal models, this problem occurs due to long simulation runtimes and is exacerbated further for stochastic models, where large simulation ensembles may be required. One approach to address this challenge is to create a simplified input–output representation of the mathematical model of interest. Such an abstraction is known as a surrogate model (also commonly referred to as a metamodel, or emulator). A surrogate model is constructed by fitting a statistical model or machine learning model to training data generated from the mathematical model of interest. Surrogate models can run several orders of magnitude faster than ABM or PDE models that they are fit to, which has motivated their application in calibration of spatiotemporal models of microbial communities [142–144]. A surrogate model enables faster exploration of the parameter space for both simulation and uncertainty analysis, albeit at the cost of relying on emulator approximation of model behavior. Although this approach is relatively new to microbial community modelling, surrogate models have been used extensively in other fields, including engineering design [145], climate simulation [146], health economics and public health [147], and ecology [148].

Surrogate models can provide significantly more efficient simulation engines when compared to the original model formulations. However, when comparing computational costs, the time required to obtain the training data for the surrogate model must be considered. The time required for training depends on several factors, such as parameter space complexity, sampling methods for parameter values, and computational cost of the original model. For example, surrogate models of an ABM for biofilm growth required approximately 1,000 hours of serial computing time (Table 2) [143,144]. In contrast, a surrogate model of a PDE model describing spatiotemporal dynamics of pattern-forming bacteria required approximately 100,000 hours of serial computing time [142]. These training times can be reduced through parallelization. For example, the actual time required to generate the training data for the works listed in Table 2 would range approximately from 1 to 7 days if 64 simulations were constantly run in parallel.

**Table 2:** Requirements for generating training data for microbial community models.

Model Type	Parameters	Simulations Required	Time per Simulation (h)	Serial Computing Time (h)	Est. Parallel Computing Time on 64 CPU cores (h)	Reference
PDE	231	100,000	0.0972	9,720	152	[142]
ABM	32	300	5–6	1,500–1,800	23–28	[143]
ABM	7	100	6–8	600–800	9–13	[144]

The use of surrogates for calibrating microbial community models requires familiarity with data sampling methods, supervised machine learning algorithms, and “big data” processing tools. Such projects may demand collaboration between data scientists and modellers who want to access these tools. Software packages, such as SUMO [149], SMT [150], and *spartan* [151], are available to facilitate the process of generating surrogate models. A brief discussion of surrogate model selection for ABM applications is provided in [152].

Surrogate models are not the only use for machine learning in this area. Lee and colleagues [153] reversed the standard modelling pipeline (of training a model on experimental data) by training a neural network to ABM data, and then using that network to infer microbial interactions from microscopy data. They demonstrated that the magnitude and direction of interspecific interactions could be quantified from steady-state spatial distributions of 2 interacting bacterial populations. Their work provides a new perspective on the use of mathematical models and machine learning to supplement experiments on microbial communities. Further advances in modelling and computer science may reveal previously unexplored features of the rich experimental data associated with single-cell observations.

## 5. Conclusions

Potential applications of microbial communities in biotechnology, health, agriculture, and energy have motivated efforts to design and manipulate both synthetic and natural communities in a predictable fashion. Predictive tools such as ABMs and PDE models will be an essential part of the microbiome engineering toolbox. Systematically calibrating microbial community models to single-cell resolution data is challenging due to the high dimensionality of the data, the intensive image processing requirements, and the specific data processing algorithms required to generate summary statistics. The study of Hartmann and colleagues [70] exemplifies how single-cell data collection and application of systematic calibration techniques can be used to predict community-level properties from single-cell behaviours.

We have compiled a collection of summary statistics relevant to microbial communities to facilitate quantitative comparison between experimental data and simulation outputs. Systematic calibration with the aid of these summary statistics can increase confidence in model predictions and overall model utility. Moreover, such calibration can improve the reusability of submodels and specific parameter values by allowing developers to confidently use or build upon these calibrated models. This modular approach is already used in synthetic circuit design workflows (e.g., [154]).

Work in macrobial ecology demonstrates how adopting standard model documentation procedures (e.g., the ODD protocol and POM framework) can result in more reproducible and therefore more useful models. Adoption of a similar standard in microbial ecology could yield similar boons in reproducibility. TDA and machine learning algorithms hold potential for facilitating systematic selection of summary statistics and efficient exploration of high-dimensional parameter spaces. As experimental methods and computational techniques continue to improve, it is expected that models will play a prominent role in rationally manipulating microbial communities in complex environments such as bioreactors, guts, soils, and wastewater treatment plants.
